# A Mendelian randomization study of alcohol use and cardiometabolic disease risk in a multi‐ancestry population from the Million Veteran Program

**DOI:** 10.1111/acer.15445

**Published:** 2024-11-24

**Authors:** Rachel L. Kember, Christopher T. Rentsch, Julie Lynch, Marijana Vujkovic, Benjamin Voight, Amy C. Justice, Million Veteran Program, Themistocles L. Assimes, Henry R. Kranzler

**Affiliations:** ^1^ Crescenz Veterans Affairs Medical Center Philadelphia Pennsylvania USA; ^2^ Department of Psychiatry University of Pennsylvania Perelman School of Medicine Philadelphia Pennsylvania USA; ^3^ Veterans Affairs Connecticut Healthcare System West Haven Connecticut USA; ^4^ Department of Internal Medicine Yale School of Medicine New Haven Connecticut USA; ^5^ Faculty of Epidemiology and Population Health London School of Hygiene & Tropical Medicine London UK; ^6^ VA Informatics and Computing Infrastructure VA Salt Lake City Health Care System Salt Lake City Utah USA; ^7^ Department of Medicine University of Pennsylvania Perelman School of Medicine Philadelphia Pennsylvania USA; ^8^ Department of Biostatistics, Epidemiology and Informatics University of Pennsylvania Perelman School of Medicine Philadelphia Pennsylvania USA; ^9^ Department of Genetics University of Pennsylvania Perelman School of Medicine Philadelphia Pennsylvania USA; ^10^ Department of Systems Pharmacology and Translational Therapeutics University of Pennsylvania Perelman School of Medicine Philadelphia Pennsylvania USA; ^11^ Institute of Translational Medicine and Therapeutics University of Pennsylvania Perelman School of Medicine Philadelphia Pennsylvania USA; ^12^ Yale School of Public Health New Haven Connecticut USA; ^13^ VA Palo Alto Health Care System Palo Alto California USA; ^14^ Department of Medicine Stanford University Stanford California USA

**Keywords:** alcohol consumption, cardiometabolic disease, Mendelian randomization, multi‐ancestry, observational study

## Abstract

**Background:**

Observational studies link moderate alcohol consumption to reduced risk of cardiometabolic diseases, including coronary heart disease (CHD) and type 2 diabetes mellitus (T2D). Mendelian randomization (MR) studies suggest that these associations are due to confounding. We present observed and genetically proxied associations between alcohol consumption and the incidence of CHD and T2D among African Americans (AA), European Americans (EA), and Hispanic Americans (HA) from the Million Veteran Program.

**Methods:**

We conducted two retrospective, nested case–control studies of 33,053 CHD and 28,278 T2D cases matched to five controls each at the time of the event (index date). We used the Alcohol Use Disorders Identification Test–Consumption (AUDIT‐C) score closest in time prior to the index date to estimate alcohol exposure. Models were adjusted for smoking, body mass index (BMI), chronic kidney disease, rheumatoid arthritis, and the use of statins or antihypertensive medications. MR analyses used either a single variant in *ADH1B* or a genetic score (GS) as instrumental variables.

**Results:**

Observational analysis showed a U‐shaped association of alcohol consumption with CHD and T2D risk. However, in MR analyses, neither *ADH1B* genotype‐predicted (in 36,465 AAs, 146,464 EAs, and 11,342 HAs) nor GS‐predicted (in EAs) alcohol consumption was associated with CHD risk. Similarly, T2D was not associated with alcohol consumption predicted either by *ADH1B* genotype (in 42,008 AAs, 109,351 EAs, and 13,538 HAs) or GS (in EAs). Multivariable MR analyses that adjusted for the effects of blood pressure and smoking also showed no association between alcohol consumption and cardiometabolic diseases.

**Conclusions:**

We replicate prior observational studies that show a U‐shaped association between alcohol consumption and cardiometabolic diseases, but MR findings show no causal association between these traits. This is largely consistent with previous MR analyses in EAs and expands the literature by providing similar findings in AA and HA populations.

## Graphical abstract

Observational studies link moderate alcohol consumption to reduced risk of cardiometabolic diseases. However, these associations may be due to confounding and potential misclassification of those who report abstinence. We conducted a retrospective and Mendelian Randomization (MR) study in individuals from the Million Veteran Program to examine the association between alcohol consumption and cardiometabolic disease. Observational analyses showed a U‐shaped association between these traits, whereas in MR analyses no association was found. Our findings confirm that previous associations are likely due to confounding.

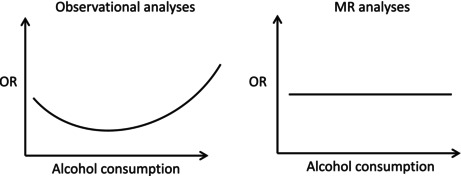

## INTRODUCTION

There is compelling evidence for an association between heavy alcohol consumption and an increased risk of coronary heart disease (CHD; Roerecke & Rehm, 2014). Although intuition would dictate that moderate alcohol consumption should also increase the risk of CHD, multiple observational studies over the last 30 years have shown a J‐ or U‐shaped association, with moderate alcohol consumption seeming to have protective effects on CHD risk in many cohorts (Krittanawong et al., 2022; Mukamal et al., 2010; Ronksley et al., 2011). Similar observational evidence exists for a detrimental effect of heavy consumption on the risk of type 2 diabetes mellitus (T2D) (Liu & Park, 2022) with a protective effect among light or moderate drinkers (Ajani et al., 2000; Baliunas et al., 2009; Djoussé et al., 2007; Koppes et al., 2005; Li et al., 2016; Liu et al., 2010).

One complicating factor in the interpretation of the health risks associated with moderate drinking is how to consider individuals who report no or low levels of recent alcohol consumption (Huth et al., 2007). This heterogeneous group of individuals includes: (i) those who stopped drinking because of health or other alcohol‐related problems; (ii) those who are lifetime abstainers; and (iii) those who falsely report abstinence or low‐level drinking (Dao et al., 2021; Gordon et al., 2020; Ng Fat, 2014). The health outcomes of each of these groups would be expected to be quite different. Moreover, the frequencies of each of these abstainer types within a cohort study (which could differ across studies of different risk populations) could lead to dramatically different relationships between the level of current alcohol consumption and risk of CHD or T2D. Confounding effects such as these can impact findings from observational studies, making it difficult to identify the true relationship between variables.

In evaluating causal relationships between moderate drinking and cardiometabolic disease, it is necessary to control for potential confounding effects of lifestyle and socioeconomic factors associated with moderate alcohol consumption, inaccurate reporting of alcohol consumption, and other, potentially unrecognized confounders. To accomplish this task, studies in the last decade have used Mendelian randomization (MR), a genotype‐predicted instrumental variable approach that controls for confounding. MR studies can be conducted as a one‐sample (i.e., conducted within the same sample) or two‐sample (conducted using summary statistics from different samples) design. A majority of studies that have used MR have shown null associations between moderate habitual use of alcohol and both CHD and T2D, with a smaller subset suggesting a detrimental effect on outcomes (Biddinger et al., 2022; Lankester et al., 2021; Liu & Park, 2022; Lu et al., 2023; Yuan & Larsson, 2020). A systematic review of these studies in general found null associations, and demonstrated that the majority of studies had been conducted in Asian or European ancestry only (van de Luitgaarden et al., 2022).

The availability of data from the Million Veteran Program (MVP) (Gaziano et al., 2016), a large, diverse sample recruited for genetic studies and linked to electronic health records (EHR), provides an opportunity to apply observational analysis and one‐sample MR in the same set of individuals to evaluate the causal association of alcohol consumption with CHD and T2D, both in all individuals and following the removal of those who report no alcohol consumption. Given that the current literature on these associations is largely limited to individuals of Asian or European ancestry, the ancestral diversity of MVP provides an opportunity to contribute additional evidence on these relationships with unprecedented power among European Americans (EAs) and initial evidence among African American (AA) and Hispanic American (HA) veterans.

## METHODS

### Study design

**FIGURE 1:**
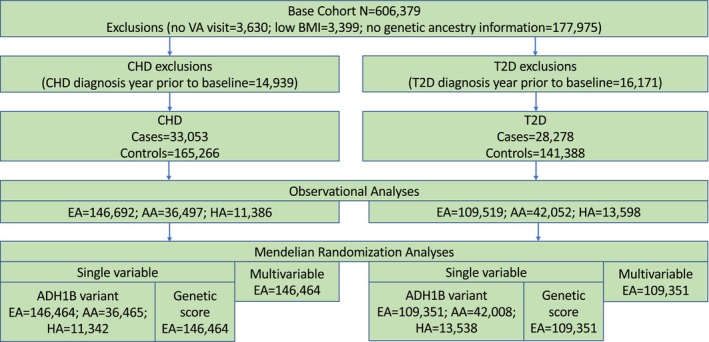
Overview of analytic datasets. Flowchart of selection from the base cohort of matched cases and controls for observational and Mendelian randomization analyses.

To examine the causal relationship between alcohol consumption and the CHD and T2D outcomes, we performed both observational and MR analyses of ΕΗRs linked to genetic data within the MVP. For the observational analyses, we conducted two parallel nested case–control studies, one for CHD and one for T2D. The analytic sample identified for this analysis was then used for MR analyses. We performed both one‐sample single variable and multivariable MR using two instruments for alcohol consumption: a single genetic variant and a genetic score (GS). Analyses were conducted separately in individuals of AA, EA, and HA ancestry. An overview of the analyses and analytic datasets is shown in Figure 1, which includes sample sizes for each of the three population groups included in the observational and MR analyses.

### Data source

The Department of Veterans Affairs (VA) healthcare system comprises more than 1200 hospitals, medical centers, and community outpatient clinics nationwide. All care is recorded in a central data repository and includes demographics, outpatient and inpatient encounters, diagnoses, smoking and alcohol consumption measures, pharmacy dispensing records, vital signs, laboratory measures, and death information.

The MVP is a mega‐biobank of veterans who accessed healthcare in the VA. The MVP received approval from the Central Veterans Affairs Institutional Review Board (IRB) and site‐specific IRBs. All MVP study participants provided written informed consent, a blood sample for genotyping, and allowed access to their EHR for research purposes.

### Study population

We first identified a base cohort of MVP patients who received VA care during the study period (October 1, 2008 through September 30, 2017, *N* = 606,379). Baseline was defined by the date on which veterans first provided a self‐reported measure of alcohol consumption that occurred at least 12 months after their first VA visit within the study period. We used the 12 months prior to the baseline date as the baseline period to ascertain baseline covariates. We excluded patients with no VA visit after baseline (*n* = 3630, 0.6%), low BMI <18.5 kg/m^2^ (*n* = 3399, 0.6%), or no genetic data (which excludes them from the MR analysis, *n* = 177,975, 29.4%). We also excluded individuals with diagnostic codes for CHD in the year prior to baseline for the CHD‐as‐outcome analysis (*n* = 14,939, 2.5%) and diagnostic codes for T2D in the year prior to baseline for the T2D‐as‐outcome analysis (*n* = 16,171, 2.7%). Each cohort was followed through the earliest incident outcome, date of death, 1 year after their last VA visit, or the end of the study period.

### Case and control selection

We extracted all inpatient and outpatient International Classification of Diseases, Ninth and Tenth revisions (ICD‐9 and ICD‐10) diagnostic codes from the VA Corporate Data Warehouse (CDW), and VA‐Fee Basis during the study period to identify cases and controls. Codes to define potential CHD cases included ICD‐9 codes 410, 411.*, 412, 414.00–414.05, 414.2, 414.3, 414.4, 414.8, 414.9, V45.81, V45.82, and ICD‐10 codes I21.*, I22.*, I23.*, I24.*, I25.1*, I25.2, I25.3, I25.5, I25.6, I25.70*, I25.71*, I25.72*, I25.73*, I25.79*, I25.810, I25.82‐I25.89, I25.9, Z95.1, and Z98.61. CHD cases were identified by the presence of one primary inpatient diagnosis, two nonprimary inpatient diagnoses, two outpatient diagnoses, two distinct problem list entry dates, or a recorded revascularization procedure. CHD controls were veterans with no CHD diagnosis, myocardial infarction diagnosis, or revascularization procedure codes.

Codes to define potential T2D cases included ICD‐9 codes 250.* (except 250.*1 and 250.*3), 357.2, 362.0, 366.41, 648.0, and ICD‐10 codes E11.* and O24.1*. T2D cases were identified by the presence of diagnoses on two distinct dates. T2D controls were those without diagnosed T2D or type 1 diabetes (ICD‐9: 250.*1, 250.*3; ICD‐10: E10.*, O24.0*) or secondary diabetes (ICD‐9: 249.*; ICD‐10: E08.*. E09.*, E13.*).

For each CHD or T2D case, we used incidence density sampling (Suissa, 2015) to match 5 controls (i.e., patients without CHD or T2D) at the time of the case event by age (±365 days), sex, race, ethnicity, baseline date (±365 days), and duration of observation. The index date was considered the date of incident CHD or T2D diagnosis for cases and the date corresponding to the same duration of time since baseline for controls. The duration of follow‐up for each patient was defined as the time between baseline and the index date. The analytic sample for CHD consisted of 33,053 cases and 165,266 controls. The analytic sample for T2D consisted of 28,278 cases and 141,388 controls.

### Alcohol exposure

We assessed alcohol consumption using the Alcohol Use Disorders Identification Test—Consumption (AUDIT‐C) questionnaire, which comprises three items that assess the quantity and frequency of alcohol use and the frequency of heavy drinking during the previous 12 months. The AUDIT‐C—the first three questions from the 10‐item AUDIT—is widely used to detect heavy drinking and/or active alcohol use disorder (Bush et al., 1998; Fiellin et al., 2000). Since 2007, the VA has required annual AUDIT‐C screening on all patients during routine healthcare visits in primary care (Bradley et al., 2006). AUDIT‐C scores range from 0 to 12, with the likelihood of physiologic injury and mortality increasing with higher AUDIT‐C scores (Justice et al., 2016). We selected the AUDIT‐C measure most proximal to diagnosis for each patient. For summary reporting in tables, we categorized AUDIT‐C scores as: 0 = abstinent, 1–3 = low, 4–7 = moderate, and ≥8 = heavy alcohol consumption.

Previous evidence shows that individuals who report no current alcohol use (AUDIT‐C = 0) are a heterogeneous group that comprises individuals who quit drinking after alcohol‐associated or other health problems, those who stopped drinking for other reasons, those who misreport abstinence, and a relatively small proportion of lifetime abstainers (Gordon et al., 2020). Thus, in the multivariable models we used AUDIT‐C = 1 as the referent group, as using individuals who report no current alcohol use as the referent group would likely cause misclassification, confounding, and weaker associations, especially among this cohort of middle‐aged and older adults.

### Covariates

We extracted information on demographics, including age at baseline, sex, and ancestry, based on the Harmonizing Genetic Ancestry and Self‐identified Race and Ethnicity (HARE) method (Fang et al., 2019). We extracted information to calculate BMI (in kg/m^2^), blood pressure category based on systolic and diastolic blood pressure (SBP and DBP), and low‐density lipoprotein (LDL) cholesterol (in mg/dL) up to 3 years prior to baseline. Blood pressure and LDL cholesterol were measured as the mean of the three closest measures prior to baseline. Blood pressure categories were defined as Normal (SBP < 120 and DBP < 80); Elevated (SBP 120–129 and DBP < 80); High, Stage 1 (SBP 130–139 or DBP 80–89); or High, Stage 2 (SBP ≥ 140 or DBP ≥ 90). Smoking status (never, former, and current) was determined by a previously validated algorithm (Song et al., 2016). We ascertained exposure to statins, antihypertensives, and corticosteroids in the year prior to baseline. The presence of chronic kidney disease or rheumatoid arthritis was determined by the presence of one inpatient or two outpatient diagnosis codes in the year prior to baseline. Information on LDL cholesterol, BMI, and smoking status was missing on 8%, 3%, and 1%, respectively, across both cohorts.

### Observational analysis

We employed unconditional logistic regression models (Pearce, 2016) to estimate odds ratios (OR) and 95% confidence intervals (CI) for the association between alcohol consumption and incident CHD or T2D. In addition to matching factors, multivariable models for both outcomes included covariates for BMI, smoking status, blood pressure category, LDL cholesterol, chronic kidney disease, rheumatoid arthritis, and exposure to statins, antihypertensives, or corticosteroids. Models using CHD as the endpoint additionally adjusted for T2D at baseline. Models using T2D as the endpoint additionally adjusted for CHD at baseline. All models were stratified by ancestry. Although MVP began recruitment in 2011, linked EHR data for all MVP participants extends as far back as 1999. Our study period began in 2008; thus, some follow‐up time included in this analysis occurred prior to participants' enrollment in MVP. Therefore, all matching procedures and analyses in this study were conditional on survival to enrollment in MVP. Analyses were performed using SAS Enterprise Guide 8.2 (SAS Institute Inc., Cary, NC, USA).

### Genotyping and imputation

Genotyping for MVP has been performed and released in batches as sample recruitment is ongoing. The analyses here were conducted with MVP Release 3 data. Genotyping was conducted using a custom Affymetrix Axiom Biobank Array. The MVP Genomics working group performed quality control and imputation (Hunter‐Zinck et al., 2020). Variants were removed if they deviated from the expected allele frequency or had a low call rate, and individuals were removed if they had a missing call rate >2.5% or excessive heterozygosity. Phasing and imputation were performed with EAGLE v2 (Loh et al., 2016) and Minimac4 (Das et al., 2016) using the 1000 Genomes (1000G) Project phase 3 (version 5) reference panel (Auton et al., 2015). We used the HARE method (Fang et al., 2019) to define ancestry groups (AA, EA, and HA).

### Instrumental variables

Our primary exposure of interest was alcohol consumption measured with the AUDIT‐C. We used two types of instrumental variables for alcohol consumption: a single variant and a genetic score created from a set of variants associated with AUDIT‐C. For single‐variant MR analyses, we used exonic variants in Alcohol dehydrogenase 1B (*ADH1B*), a gene whose association with alcohol use is well established. In AAs, the variant most significantly associated with alcohol use is rs2066702 (Arg370Cys), while in EAs and Has, it is rs1229984 (Arg47His) (Kranzler et al., 2019).

To generate an instrumental variable using multiple single nucleotide polymorphisms (SNPs) associated with alcohol consumption, we identified a set of variants associated with AUDIT‐C in an independent sample—the UK Biobank (*N* = 121,604) (Sanchez‐Roige et al., 2019)—and used them to construct a genetic risk score (GS). We selected genome‐wide significant variants to increase the likelihood that they were truly associated with the exposure and therefore met the first assumption of MR. There were 1800 variants that were genome‐wide significant (*p* < 5 × 10^−8^) in the UK Biobank, and although rs1229984 was excluded from the summary statistics because it failed a Hardy–Weinberg Equilibrium test (Sanchez‐Roige et al., 2019), we included it in the generation of the GS given its strength as an instrumental variable, using the effect size as reported in supplementary tables for that study. We limited the variant set to those present in MVP (*N* = 1263 in EAs and 1593 in AAs). We did not construct a GS in HAs due to the smaller sample size. After merging with variants present in MVP, each set of variants was clumped using a range of 500 kb, *r* ^2^ > 0.1, and the 1000G European ancestry reference panel, leaving a set of 9 variants in the AA dataset and 10 variants in the EA dataset. GS for each individual were constructed using the ‐score function in PLINK (Chang et al., 2015).

To calculate genetic scores for blood pressure and smoking in EAs, we selected sets of variants with prior associations with high blood pressure (HBP) (Zhu et al., 2019) and smoking initiation (Liu et al., 2019). We selected variants that were genomewide significant in these GWAS and present in the MVP sample (*N* = 9223 SNPs for HBP and *N* = 3873 SNPs for smoking). Following clumping (range of 500 kb, *r* ^2^ > 0.1) based on 1000G European ancestry, 380 SNPs remained for HBP and 102 SNPs remained for smoking. GS were constructed using the ‐score function in PLINK.

### Mendelian randomization analyses

**FIGURE 2:**
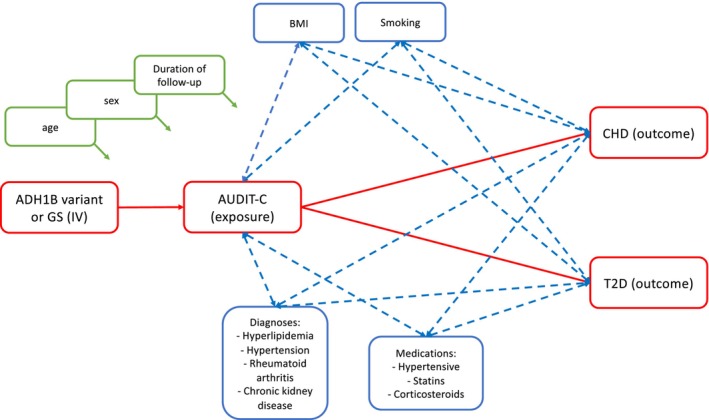
Overview of relationship between variables in MR analysis. Red boxes denote the variables and paths that we are testing for causality. Green boxes denote covariates included in the model. Blue boxes denote potential confounders of the relationship between AUDIT‐C and CHD or T2D.

We performed one‐sample MR analyses to estimate the causal effect of alcohol consumption on CHD and T2D. First, we assessed the association between each instrumental variable (SNP or GS) and the exposure (AUDIT‐C) using linear regression and calculated the *F*‐statistic to test the strength of the instrument. Next, we tested the association between each instrumental variable (SNP or GS) and potential confounders (BMI, SBP, LDL cholesterol, current smoking (binary; current vs. former/never/unknown), exposure to statins (binary), exposure to antihypertensives (binary), exposure to corticosteroids (binary), chronic kidney disease (binary), and rheumatoid arthritis (binary); see Figure 2) using logistic or linear regression as appropriate. We considered confounders significant at a Bonferroni‐corrected *p*‐value <0.0056. We conducted single variable MR with the 2‐stage least‐squares (2SLS) method using the “ivpack” package in R. Given the significant association between the GS instrumental variable and SBP and smoking in EAs, we also performed multivariable MR using instrumental variables for HBP and smoking. We conducted analyses within each ancestry in the total sample and also following the removal of AUDIT‐C = 0 given this group's heterogeneity (Dao et al., 2021; Gordon et al., 2020). All MR analyses included age, sex, and duration of follow‐up as covariates.

## RESULTS

### Cohort characteristics

To evaluate the relationship between alcohol consumption and CHD and T2D risk, we first developed a base cohort in MVP (*N* = 606,379). Application of exclusion criteria (listed in methods) yielded 311,933 patients eligible for matching in the CHD cohort and 254,471 patients in the T2D cohort (Figure 1). Of 33,123 patients who received an incident CHD diagnosis in the CHD cohort, 33,053 (99.8%) were matched to five controls and included in the case–control analysis. Of the 28,326 patients with an incident T2D diagnosis in the T2D cohort, 28,278 (99.8%) were matched to five controls and included in the case–control analysis. Of the 267,912 controls across the CHD and T2D analyses, 63,002 (23.5%) appeared in both analyses.

The median age of the 198,319 patients in the CHD analysis was 61 years, with 97% male, 74% EA, 18% AA, 6% HA, and a median follow‐up of 3.2 years. These matching characteristics did not differ by case/control status. In this cohort, 35.8% cases and 38.1% controls reported low alcohol consumption, 10.7% cases and 12.9% controls reported moderate alcohol consumption, and 2.7% cases and 2.5% controls reported heavy alcohol consumption proximal to the index date (Table 1). In the T2D analysis, the median age of the 169,666 patients was 59 years, with 93% male, 65% EA, 25% AA, and 8% HA, and the median follow‐up was 2.7 years. In this cohort, 37.4% cases and 39.4% controls reported low alcohol consumption, 9.9% cases and 13.6% controls reported moderate alcohol consumption, and 2.8% cases and 3.3% controls reported heavy alcohol consumption proximal to the index date (Table 1). Approximately half of the CHD and T2D cohorts reported no alcohol consumption proximal to the index date.

**TABLE 1:** Characteristics of incident coronary heart disease and incident type 2 diabetes cases and matched controls.

	CHD		T2D	
	Cases	Controls	Cases	Controls
	*n* = 33,053	*n* = 165,266	*n* = 28,278	*n* = 141,388
Matching variables				
Age, years, median (IQR)	61 (57–67)		59 (52–64)	
Male sex, %	97%		93%	
Race and ethnicity, %				
European‐American	74%		65%	
African‐American	18%		25%	
Hispanic‐American	6%		8%	
Other/unknown	2%		3%	
Person‐years, median (IQR)	3.2 (1.4–5.5)		2.7 (1.1–4.8)	
Proximal exposure				
Alcohol consumption				
Abstinent	16,803 (50.8)	77,041 (46.6)	14,093 (49.8)	61,791 (43.7)
Low	11,816 (35.8)	62,901 (38.1)	10,588 (37.4)	55,695 (39.4)
Moderate	3549 (10.7)	21,234 (12.9)	2808 (9.9)	19,177 (13.6)
Heavy	885 (2.7)	4090 (2.5)	789 (2.8)	4725 (3.3)
Baseline covariates				
Body mass index, kg/m^2^				
<25	4633 (14.0)	28,179 (17.1)	2260 (8.0)	29,247 (20.7)
25–29.9	11,532 (34.9)	64,561 (39.1)	8321 (29.4)	57,340 (40.6)
≥30	16,419 (49.7)	70,862 (42.9)	17,138 (60.6)	52,796 (37.3)
Missing	469 (1.4)	1664 (1.0)	559 (2.0)	2005 (1.4)
Smoking status				
Current	9588 (29.0)	41,004 (24.8)	8495 (30.0)	43,119 (30.5)
Former	15,972 (48.3)	78,920 (47.8)	12,767 (45.2)	60,451 (42.8)
Never	7152 (21.6)	43,383 (26.3)	6719 (23.8)	36,227 (25.6)
Missing	341 (1.0)	1959 (1.2)	297 (1.1)	1591 (1.1)
Blood pressure				
Normal	4649 (14.1)	27,860 (16.9)	4277 (15.1)	28,375 (20.1)
Elevated	5877 (17.8)	31,834 (19.3)	4738 (16.8)	25,812 (18.3)
High, Stage 1	16,178 (49.0)	81,155 (49.1)	14,291 (50.5)	67,929 (48.0)
High, Stage 2	6349 (19.2)	24,417 (14.8)	4972 (17.6)	19,272 (13.6)
LDL cholesterol, mg/dL				
<70	2816 (8.5)	12,782 (7.7)	2097 (7.4)	8814 (6.2)
70–99	9632 (29.1)	48,144 (29.1)	7366 (26.1)	36,306 (25.7)
100–129	10,931 (33.1)	57,941 (35.1)	9556 (33.8)	50,015 (35.4)
≥130	7393 (22.4)	34,892 (21.1)	6959 (24.6)	34,954 (24.7)
Missing	2281 (6.9)	11,507 (7.0)	2300 (8.1)	11,299 (8.0)
Type 2 diabetes	5838 (17.7)	23,530 (14.2)		
Coronary heart disease			5683 (20.1)	20,353 (14.4)
Chronic kidney disease	2602 (7.9)	7787 (4.7)	1111 (3.9)	4893 (3.5)
Rheumatoid arthritis	566 (1.7)	2382 (1.4)	364 (1.3)	2129 (1.5)
Statins	15,404 (46.6)	66,516 (40.3)	11,495 (40.7)	47,781 (33.8)
Antihypertensives	22,189 (67.1)	94,868 (57.4)	17,204 (60.8)	71,993 (50.9)
Corticosteroids	9432 (28.5)	42,154 (25.5)	7816 (27.6)	36,194 (25.6)

*Note*: All statistics reported as *n* (%) unless otherwise stated; up to five controls were matched to each case on age, sex, race, ethnicity, person‐time, and baseline date.

Abbreviations: CHD, coronary heart disease; IQR, interquartile range; LDL, low density lipoprotein; T2D, type 2 diabetes.

### Case–control analysis

**FIGURE 3:**
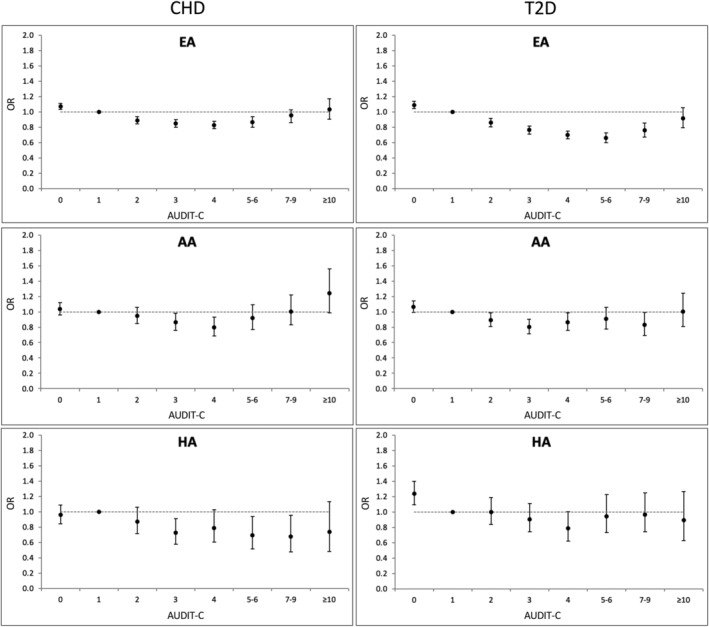
Observed associations between alcohol consumption and incident CHD or type 2 diabetes. Odds ratios (OR) were calculated from individual logistic regression models comparing each AUDIT‐C score in turn against AUDIT‐C = 1 as the referent group.

In fully adjusted models, we observed nonlinear associations between alcohol consumption and both CHD and T2D. For both EA and AA patients, the odds of incident CHD or T2D followed a general U‐shaped pattern. Low‐to‐moderate alcohol consumption was associated with lower odds for both outcomes, while the lowest and highest AUDIT‐C scores showed null or positive associations (Figure 3; Table S1). This pattern of association was not clearly observed among HA patients, likely due to the smaller sample size and wide confidence intervals in this population group.

### MR analysis

Genotype frequencies of rs1229984 (Arg47His) and rs2066702 (Arg370Cys) are reported in Table S2. The presence of the *ADH1B* variant alleles (i.e., rs1229984‐T and rs2066702‐A) corresponded with lower alcohol consumption as measured by mean AUDIT‐C score (Table S3). The *ADH1B* variants were significantly associated with lower AUDIT‐C scores (Tables 2 and 3) and a strong instrumental variable in all ancestry groups as measured by the *F*‐statistic. The GS for AUDIT‐C was significantly associated with greater AUDIT‐C scores in EAs (Tables 2 and 3), but not in AAs (CHD: beta = 0.005, SE = 0.011, *p* = 0.639; T2D: beta = 0.021, SE = 0.011, *p* = 0.064). For MR analyses, we selected as instrumental variables the *ADH1B* variant and the GS in EAs and the *ADH1B* variant in AAs and HAs.

**TABLE 2:** Association of instrumental variable with outcome and confounders in the coronary heart disease dataset.

	European American				African American		Hispanic American	
	*ADH1B* SNP		Genetic score		*ADH1B* SNP		*ADH1B* SNP	
*F*‐statistic	360.6				256		51.25	
	**Beta**	***p***	**Beta**	***p***	**Beta**	***p***	**Beta**	***p***
AUDIT‐C score	−0.26 (0.02)	5.23 × 10^−41^	0.08 (0.01)	2.99 × 10^−50^	−0.12 (0.02)	3.85 × 10^−10^	−0.39 (0.06)	3.89 × 10^−11^
Body Mass Index	−0.29 (0.05)	8.69 × 10^−8^	−0.001 (0.01)	0.946	0.07 (0.05)	0.201	−0.19 (0.15)	0.206
Hyperlipidemia (LDL)	−0.45 (0.29)	0.118	0.12 (0.08)	0.121	−0.47 (0.31)	0.13	−1.78 (0.86)	0.038
Blood pressure (SBP)	−1.28 (0.13)	2.54 × 10^−23^	0.37 (0.04)	5.59 × 10^−26^	0.19 (0.14)	0.169	−0.72 (0.36)	0.048
	**OR (95% CI)**	***p***	**OR (95% CI)**	***p***	**OR (95% CI)**	***p***	**OR (95% CI)**	***p***
Coronary heart disease	0.96 (0.91–1.01)	0.113	1.00 (0.99–1.01)	0.964	0.99 (0.94–1.04)	0.608	0.90 (0.77–1.04)	0.141
Smoking (current)	0.98 (0.93–1.02)	0.323	1.03 (1.01–1.04)	3.34 × 10^−5^	1.01 (0.97–1.05)	0.511	1.04 (0.92–1.18)	0.534
Rheumatoid arthritis	0.99 (0.85–1.14)	0.853	1.03 (0.99–1.07)	0.191	0.93 (0.78–1.12)	0.458	0.64 (0.37–1.12)	0.116
Chronic kidney disease	1.13 (1.04–1.22)	0.005	0.97 (0.95–1.00)	0.023	1.03 (0.96–1.10)	0.456	0.94 (0.73–1.21)	0.634
Meds: Hypertensive	0.89 (0.86–0.93)	1.24 × 10^−8^	1.02 (1.01–1.04)	6.53 × 10^−6^	0.98 (0.94–1.02)	0.394	0.90 (0.80–1.00)	0.05
Meds: Statins	0.97 (0.94–1.01)	0.187	1.00 (0.99–1.01)	0.473	1.00 (0.96–1.04)	0.928	0.97 (0.87–1.08)	0.542
Meds: Corticosteroids	0.99 (0.94–1.03)	0.525	1.00 (0.99–1.01)	0.987	1.00 (0.96–1.04)	0.977	0.93 (0.82–1.05)	0.223

Abbreviations: AUDIT‐C, Alcohol Use Disorders Identification Test‐Consumption; LDL, low‐density lipoprotein cholesterol; SBP, systolic blood pressure.

**TABLE 3:** Association of instrumental variable with outcome and cofounders in the type 2 diabetes dataset.

	European American				African American		Hispanic American	
	*ADH1B* SNP		Genetic score		*ADH1B* SNP		*ADH1B* SNP	
*F*‐statistic	333.1				249.4		73.79	
	**Beta**	***p***	**Beta**	***p***	**Beta**	***p***	**Beta**	***p***
AUDIT‐C score	−0.33	<2 × 10^−16^	0.093	<2 × 10^−16^	−0.15 (0.02)	3.63 × 10^−14^	−0.47 (0.06)	4.32 × 10^−16^
Body Mass Index	−0.19	0.002	−0.004	0.789	−0.03 (0.05)	0.589	−0.14 (0.13)	0.276
Hyperlipidemia (LDL)	−0.52	0.134	0.15	0.114	−0.72 (0.30)	0.015	−1.84 (0.78)	0.018
Blood pressure (SBP)	−0.88	9.26 × 10^−9^	0.28	2.21 × 10^−11^	−0.04 (0.13)	0.743	−0.44 (0.32)	0.169
	**OR (95% CI)**	***p***	**OR (95% CI)**	***p***	**OR (95% CI)**	***p***	**OR (95% CI)**	***p***
Type 2 diabetes	0.95 (0.89–1.01)	0.082	1.00 (0.99–1.02)	0.786	1.02 (0.97–1.07)	0.475	0.90 (0.79–1.03)	0.126
Smoking (current)	0.93 (0.89–0.99)	0.012	1.03 (1.02–1.05)	1.70 × 10^−6^	1.03 (1.00–1.07)	0.085	1.03 (0.93–1.15)	0.558
Rheumatoid arthritis	1.01 (0.85–1.20)	0.93	1.00 (0.96–1.05)	0.937	1.27 (1.09–1.48)	0.002	1.32 (0.91–1.92)	0.144
Chronic kidney disease	1.18 (1.05–1.32)	0.004	0.96 (0.93–0.99)	0.006	1.07 (0.98–1.16)	0.127	0.81 (0.58–1.13)	0.211
Meds: Hypertensive	0.93 (0.89–0.97)	0.002	1.01 (1.00–1.02)	0.181	0.98 (0.94–1.01)	0.237	1.02 (0.92–1.13)	0.729
Meds: Statins	1.00 (0.96–1.05)	0.899	0.99 (0.97–1.00)	0.036	0.98 (0.94–1.02)	0.405	0.87 (0.77–0.97)	0.0128
Meds: Corticosteroids	0.95 (0.90–1.00)	0.045	1.00 (0.99–1.02)	0.687	1.02 (0.98–1.06)	0.248	1.07 (0.96–1.20)	0.208

Abbreviations: AUDIT‐C, Alcohol Use Disorders Identification Test‐Consumption; LDL, low‐density lipoprotein cholesterol; SBP, systolic blood pressure.

Single‐variable MR analyses did not yield evidence of a causal role for alcohol consumption in susceptibility to CHD or T2D across the instrumental variables used and ancestry groups (all *p* > 0.05; Table 4). In sensitivity analyses that excluded individuals who reported no alcohol consumption (i.e., AUDIT‐C = 0), greater alcohol consumption nominally increased susceptibility to T2D in EAs in the analysis focused on the *ADH1B* SNP instrumental variable (OR = 1.02, *p* = 0.018). The analysis that used the GS as an instrumental variable was directionally consistent and showed a nonsignificant trend (OR = 1.02, *p* = 0.071; Table 4).

**TABLE 4:** Single variable Mendelian randomization in the coronary heart disease (CHD) and type 2 diabetes (T2D) datasets for European Americans, African Americans, and Hispanic Americans.

	European American				African American		Hispanic American	
	*ADH1B* SNP		Genetic score		*ADH1B* SNP		*ADH1B* SNP	
	OR (95% CI)	*p*	OR (95% CI)	*p*	OR (95% CI)	*p*	OR (95% CI)	*p*
All								
CHD	1.02 (1.00–1.05)	0.117	1.00 (0.98–1.02)	0.964	1.01 (0.96–1.07)	0.609	1.04 (0.99–1.09)	0.154
T2D	1.02 (1.00–1.05)	0.082	1.00 (0.98–1.03)	0.787	0.98 (0.94–1.03)	0.476	1.03 (0.99–1.07)	0.136
Removing AUDITC = 0								
CHD	1.01 (0.99–1.03)	0.293	1.00 (0.98–1.02)	0.798	1.01 (0.97–1.06)	0.556	1.03 (0.99–1.08)	0.175
T2D	1.02 (1.00–1.04)	0.018	1.02 (1.00–1.03)	0.071	0.97 (0.94–1.01)	0.118	1.00 (0.96–1.04)	0.992

However, both instrumental variables were significantly associated with confounders in EAs (*ADH1B* variant: BMI, SBP, hypertensive medications; GS: SBP, smoking, hypertensive medications; Tables 2 and 3), which violates a key assumption of MR. To address this violation, we constructed instrumental variables for smoking and high blood pressure (HBP) and conducted a multivariable MR analysis. The GS for smoking was significantly associated with current smoking and was a strong instrumental variable (Table S4). The GS for HBP was significantly associated with SBP and was also a strong instrumental variable (Table S4). In single‐variable MR analyses, blood pressure, but not smoking, was positively causally associated with CHD and T2D (Table S4). Multivariable MR analyses (Table 5) did not yield evidence of an association of alcohol consumption with either CHD or T2D when adjusting for blood pressure and smoking. However, blood pressure remained positively associated with CHD and T2D when adjusting for alcohol consumption and smoking, an effect that remains significant when removing individuals with AUDIT‐C = 0.

**TABLE 5:** Multivariable MR analysis for AUDIT‐C, smoking, and blood pressure.

	CHD		T2D	
	OR (95% CI)	*p*	OR (95% CI)	*p*
All				
AUDIT‐C score	0.97 (0.93–1.00)	0.054	0.97 (0.92–1.01)	0.133
Blood pressure (SBP)	1.01 (1.00–1.01)	4.63 × 10^−4^	1.00 (1.00–1.02)	0.005
Smoking (current)	0.99 (0.78–1.27)	0.961	1.16 (0.79–1.71)	0.443
Removing AUDIT‐C = 0				
AUDIT‐C	0.96 (0.93–1.00)	0.051	0.99 (0.95–1.04)	0.686
Blood pressure (SBP)	1.01 (1.00–1.01)	0.042	1.01 (1.00–1.02)	0.047
Smoking (current)	1.15 (0.82–1.61)	0.406	1.07 (0.68–1.70)	0.765

## DISCUSSION

In this study, we examined associations of alcohol consumption—measured as an AUDIT‐C score—with risk of CHD and T2D. Consistent with prior literature (Mukamal et al., 2010; Ronksley et al., 2011), observational analysis showed a U‐shaped relationship for both outcomes in both AA and EA strata, with the risk for both CHD and T2D lowest among individuals with low‐to‐moderate AUDIT‐C scores. This pattern of association was not seen among the smaller group of HAs, which was only 7.8% the size of the EA sample and 31.1% that of the AA sample. This contrasts with the EA sample, which was approximately four times the size of the AA sample and had the smallest confidence intervals and the most obvious U‐shaped relationship between AUDIT‐C score and risk of both cardiometabolic traits. These findings underscore the need for larger samples of non‐European populations to evaluate these associations further.

With the application of MR to account for unmeasured confounding, in the full sample we found no evidence that drinking level was causal for either CHD or T2D in AAs, EAs, or HAs. This lack of a causal effect was evident when using as an instrumental variable either a single SNP—*ADH1B*—or a GS comprised of variants from an independent genome‐wide association study of AUDIT‐C (Sanchez‐Roige et al., 2019). Population‐specific nonsynonymous substitutions in *ADH1B* have consistently been identified in genome‐wide association studies of both alcohol consumption and AUD across multiple populations, with the SNPs generally being the most significant findings (Clarke et al., 2017; Deak et al., 2022; Kranzler et al., 2019; Lai et al., 2019; Liu et al., 2019; Matoba et al., 2020; Sanchez‐Roige et al., 2019; Walters et al., 2018; Zhou et al., 2022). In both univariate and multivariable models, the population‐specific *ADH1B* variant alleles were associated with lower alcohol consumption and were a strong instrumental variable in all ancestry groups.

Individuals with AUDIT‐C = 0 (i.e., those reporting no alcohol consumption during the preceding year—the timeframe over which AUDIT‐C queries alcohol consumption) are a heterogeneous group. Current abstainers comprise individuals who stopped drinking because of health or other alcohol‐related problems, those who may falsely report abstinence or low‐level drinking, and lifetime abstainers (Dao et al., 2021; Ng Fat, 2014). In within‐ancestry analyses that followed removal of AUDIT‐C = 0, we found alcohol consumption to be positively associated with T2D in EAs when using the *ADH1B* SNP as the instrumental variable and a nonsignificant trend for association when using the GS as the instrumental variable. However, it should be noted that the *ADH1B* SNP was associated with BMI, which may have acted as a confounder in these analyses. In multivariable MR analyses using the GS that include instrumental variables for smoking and blood pressure, this causal association was no longer seen, suggesting that its presence may have been due to additional confounding by these variables. We conclude from these findings that the observational associations showing a U‐shaped curve for the association of drinking level with CHD and T2D reflect confounding attributable to a variety of unmeasured factors. These include inaccurate or invalid reporting of alcohol consumption and lifestyle, genetic, and socioeconomic factors associated with moderate alcohol consumption.

Limitations of this study include large differences in the size of the three population groups, which limit cross‐ancestry comparisons of the effects of drinking level on cardiometabolic disease risk. While the instrumental variables in all ancestries had *F*‐statistics greater than 10, their strength varied, with EAs and AAs having much larger *F*‐statistics than HAs. Second, another potential effect due to the larger EA sample is that both the *ADH1B* SNP and the GS were significantly associated with confounders, which violates an assumption of MR. To address this, in the EA sample we constructed instrumental variables for smoking and blood pressure—which we found to be strong instrumental variables—for use in multivariable MR analysis, which controlled the potential confounding. Third, we assessed alcohol consumption proximal to the diagnosis of CHD and T2D to minimize the potential for misclassification of alcohol exposure at the time of the event. Although the AUDIT‐C captures alcohol exposure over the previous 12 months, a single AUDIT‐C measure may not reflect longer‐term patterns of alcohol consumption. Fourth, the available data did not allow for distinctions between, for example, lifetime abstainers and individuals who abstained during the preceding year due to adverse effects of drinking or non‐alcohol‐related medical illness (Gordon et al., 2020). However, in secondary analyses, we accounted for biases introduced by such heterogeneity in the abstainer group by removing this group. Fifth, our study period included follow‐up from 2008, while MVP enrollment began in 2011. Thus, for some patients, inclusion in this study was conditional on survival to enrollment into MVP. Given the high morbidity and mortality rate associated with CHD, selection bias could have impacted results. Sixth, while patients in VA care represent a diversity of backgrounds, women represented a small proportion of individuals in the sample, potentially limiting the generalizability of our results.

Despite these limitations, ours is the largest study to date of AA and HA populations that evaluated the relations between drinking level and either CHD or T2D. Our results suggest that, as has been shown in larger population groups, there are not beneficial effects of moderate alcohol consumption on cardiometabolic disease. Additional studies using MR in non‐European samples are needed to confirm these initial results.

## FUNDING INFORMATION

This work was supported by Merit Review Awards from the US Department of Veterans Affairs Biomedical Laboratory Research and Development Service (no. I01 BX003341 (to A.C.J. and H.R.K.)); the VISN 4 Mental Illness Research, Education, and Clinical Center (to H.R.K.); and NIAAA grant K01 AA028292 (to R.L.K.). The funders had no role in study design, data collection and analysis, decision to publish, or preparation of the manuscript. The views expressed in this article are those of the authors and do not necessarily represent the position or policy of the Department of Veterans Affairs or the US Government.

## CONFLICT OF INTEREST STATEMENT

Dr. Kranzler is a member of advisory boards for Altimmune, Clearmind Medicine, Dicerna Pharmaceuticals, Enthion Pharmaceuticals, and Sophrosyne Pharmaceuticals; a consultant to Sobrera Pharmaceuticals; the recipient of research funding and medication supplies for an investigator‐initiated study from Alkermes; a member of the American Society of Clinical Psychopharmacology's Alcohol Clinical Trials Initiative, which was supported in the last 3 years by Alkermes, Dicerna, Ethypharm, Lundbeck, Mitsubishi, Otsuka, and Pear Therapeutics; and a holder of U.S. patent 10900082 titled “Genotype‐guided dosing of opioid agonists,” issued 26 January 2021.

## Supporting information


Tables S1‐S4

## Data Availability

Data sharing is not applicable to this article as no new data were created or analyzed in this study.
